# Roux-en-Y duodenojejunostomy improves gastric emptying in experimental obstruction of the distal duodenum

**DOI:** 10.1371/journal.pone.0199759

**Published:** 2018-06-28

**Authors:** Sławomir Mrowiec, Krzysztof Jonderko, Zygmunt Górka, Paweł Lampe, Anna Kasicka-Jonderko, Joanna Kołodziejczak-Nalewajka, Katarzyna Kuśnierz, Marek Olakowski

**Affiliations:** 1 Department of Gastrointestinal Surgery, School of Medicine, Medical University of Silesia, Katowice, Poland; 2 Department of Basic Biomedical Science, School of Pharmacy, Medical University of Silesia, Sosnowiec, Poland; 3 Department of Radiodiagnostics and Nuclear Medicine, School of Medicine, Medical University of Silesia, Katowice, Poland; Shanghai Diabetes Institute, CHINA

## Abstract

**Introduction:**

We undertook a comparative survey of gastric emptying (GE) kinetics after two variants of bypass surgery for upper bowel obstruction.

**Material & methods:**

In 10 dogs with experimental upper bowel obstruction, five were randomized to obtain gastrojejunal anastomosis (GA), and the other five received Roux-en-Y duodenojejunal anastomosis (DA). Duplicate scintigraphic measurements of GE of a solid meal were accomplished in every animal before surgery and during the early (2–3 weeks), medium (3 months), and late (6 months) post-operative period. The GE curves were fitted with a power-exponential function to derive the GE half time T½, and the curve shape parameter S.

**Results:**

Early after surgery T½ slightly decreased by -18±21 min in the DA group and lengthened by 91±37 min in the GA group (p = 0.042). In both groups an increase in the S parameter was found then. In either group T½ gradually declined towards the basal value during the medium and late post-operative period. On the other hand, net differences relative to the basal situation in the S values appeared to be positive in the GA group (0.32±0.11 at 3 months; 0.64±0.19 at six months), and negative in the DA group (-0.30±0.09 at 3 months; -0.01±0.20 at six months). Hence a statistically significant contrast was found between those differences: p = 0.0022 at 3 months, and p = 0.045 at six months after the surgery.

**Conclusion:**

Roux-en-Y duodenojejunal anastomosis appears to be superior to the classical gastrojejunal anastomosis while restoring patency of the gastrointestinal passage in the case of upper bowel obstruction.

## Introduction

Cancer of the corpus and/or of the caudal part of the pancreas rarely causes jaundice but infiltrates the celiac plexus causing abdominal pain. Subsequently, infiltration of the horizontal and ascending part of the duodenum leads to upper bowel obstruction [1]. It has also been confirmed that during the later stages of pancreatic cancer 8–50% of patients develop obstruction of the duodenum. At the moment of diagnosis as many as 30–50% of them develop symptoms of upper digestive tract obstruction including nausea and vomiting [2].

Barkin et al. [3] examined gastric emptying in patients with pancreatic cancer associated with an obstruction of the duodenum. The study revealed that 60% of the patients exhibited abnormal gastric emptying. Theoretically, resective procedures should be the optimal choice in comparison to palliative procedures. In real life, however, a low survival rate after unsuccessful resections implies the use of palliative procedures as an alternative. Therefore, a palliative surgical procedure should be considered a primary option in the case of every exploratory laparotomy revealing an unresectable tumor [4].

In 1881 Wölfler conducted the first gastro-jejunal anastomosis in a patient with pyloric cancer [5]. Later on, Coller and Winfield in 1934 [6], as well as many other followers, searched for the most effective palliative operative procedure for unresectable pancreatic malignant tumors [7–10]. The most common complications, frequently observed after a gastrojejunal anastomosis, include nausea, gastric “fullness”, persisting vomiting, hemorrhage from the upper part of the alimentary canal, duodenogastric reflux, regurgitation of the contens of the duodenum to the stomach, alkaline-induced gastritis and gastric ulcers [11–14].

Surgical reconstruction of the alimentary canal, using the superior part of the duodenum, was the subject of several experimental procedures [15,16]. Because reconstructive procedures for a bowel obstruction at the level of the inferior part of the duodenum with the use of a gastrojejunal anastomosis proved to be unsatisfactory, it seems imperative to work out a procedure preserving the pylorus which would allow a physiological bowel passage [12,17].

This study aimed at a survey of gastric emptying kinetics at various post-surgery periods, determined non-invasively with the use of scintigraphy, in dogs supplied with a gastrojejunal or Roux-en-Y duodenojejunal anastomosis created after an experimental upper bowel obstruction.

## Material and methods

**Fig 1:**
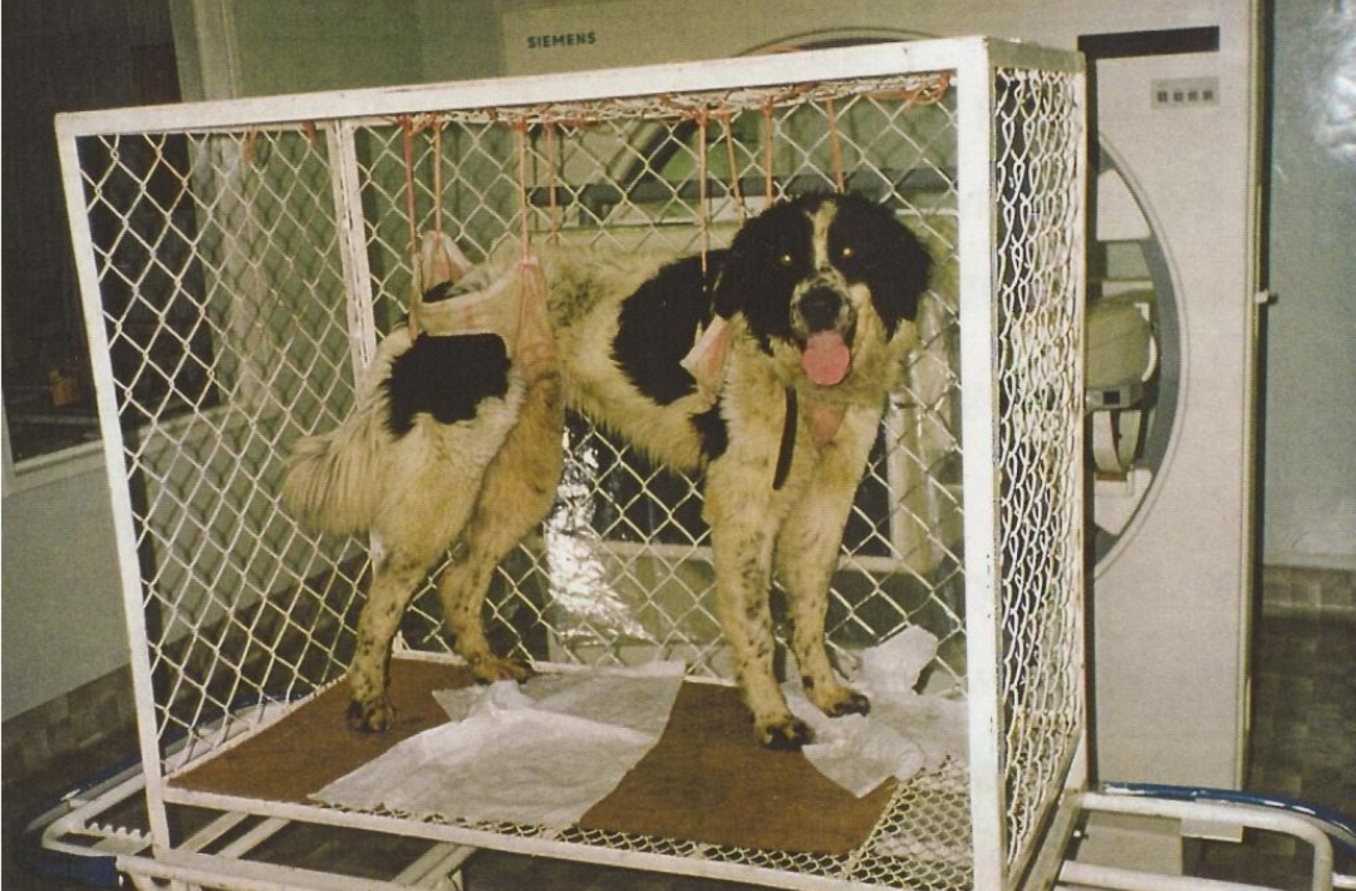
Modified Pavlov stand: The head of a gamma camera is placed close to the left side of the dog.

The project was approved by the Bioethical Committee of the Medical University of Silesia. It was conducted on 10 mongrel dogs weighing on average 21.5 kg (range: 18–26 kg) which were obtained from the Center of Experimental Medicine at the Medical University of Silesia. For 14 days preceding the start of the examinations the dogs were trained to become accustomed to a modified Pavlov stand. The stand was constructed in order to enable a proper acquisition of scintigraphic images from the left lateral projection of the stomach of in an animal remaining in a standing position (Fig 1). The dogs were given their meals every day while being placed in the stand, and were trained to remain still therein for 3 hours. Thereafter, a basal preoperative measurement of gastric emptying was taken in every dog twice at a one-week interval. During the rest of the day the dogs were kept in individual cages, where they had an ad libitum access to water. In the premises a constant temperature of 20 degrees Celsius was maintained, and a 12 by 12-hour day and night lighting cycle was kept.

The dogs were next randomly assigned to two groups, each consisting of five animals.

After an overnight fast, a dog was premedicated with an initial intravenous infusion of 5 mg/kg sodium thiopental. Subsequently inhalation anaesthesia was maintained with 1.5% isoflurane in oxygen–nitrous oxide (1:1) carrier gases delivered from a ventilator following endotracheal intubation.

**Fig 2:**
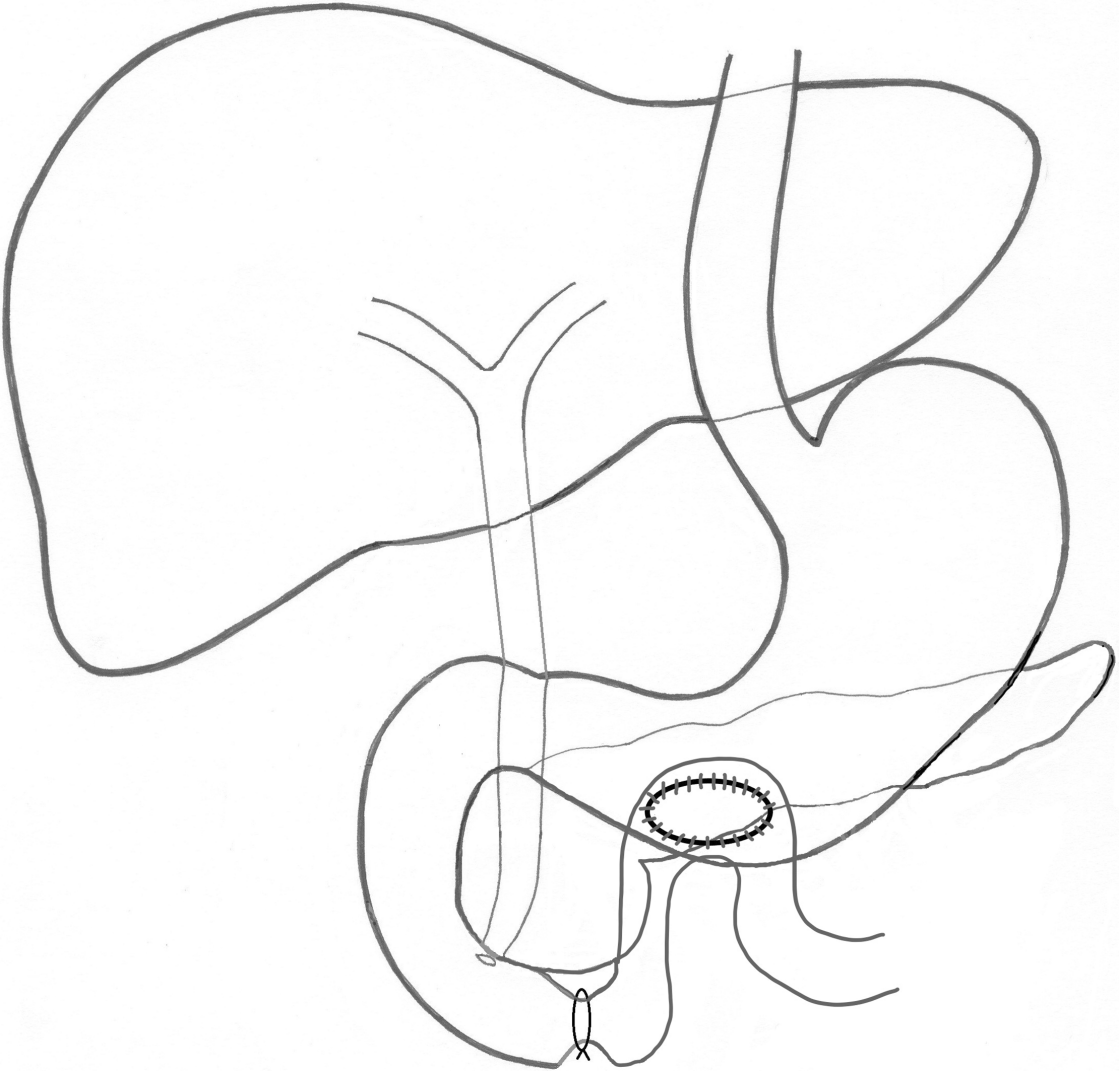
Draft of the post-surgery anatomical situation in dogs provided with a gastroanastomosis.

In the first group of dogs, the gastroanastomosis (GA) was performed. After opening the abdominal cavity in the dog, typically a bloated, hook-shaped stomach will be exposed, which is directed transversely to the long axis of the animal. Therefore, attention must be paid to make the anastomosis in the lowest part of the dog's stomach, so that it would empty freely. In the first step, an incomplete small bowel obstruction was created at the lower part of the duodenum. Then a gastrojejunal anastomosis by-pass was performed above the site of the obstruction. Accordingly, the stomach was opened through the anterior wall at the border of the body and the antrum, and herein an 8 mm catheter was temporarily introduced into the lumen of the duodenum. An occluding double ligature (PDS # 5, Ethicon, U.K.) was placed around the catheter bringing about an artificial duodenal obstruction to the diameter of the catheter. Upon removal of the catheter a side-to-side gastrojejunal anastomosis was created (PDS 4–0, Ethicon, U.K.) passing the intestine extracolically. The abdominal integument was sutured with single sutures in each layer and wound-dressing was applied. The sketch of the resultant post-surgery anatomical situation in the GA dogs is provided in Fig 2.

**Fig 3:**
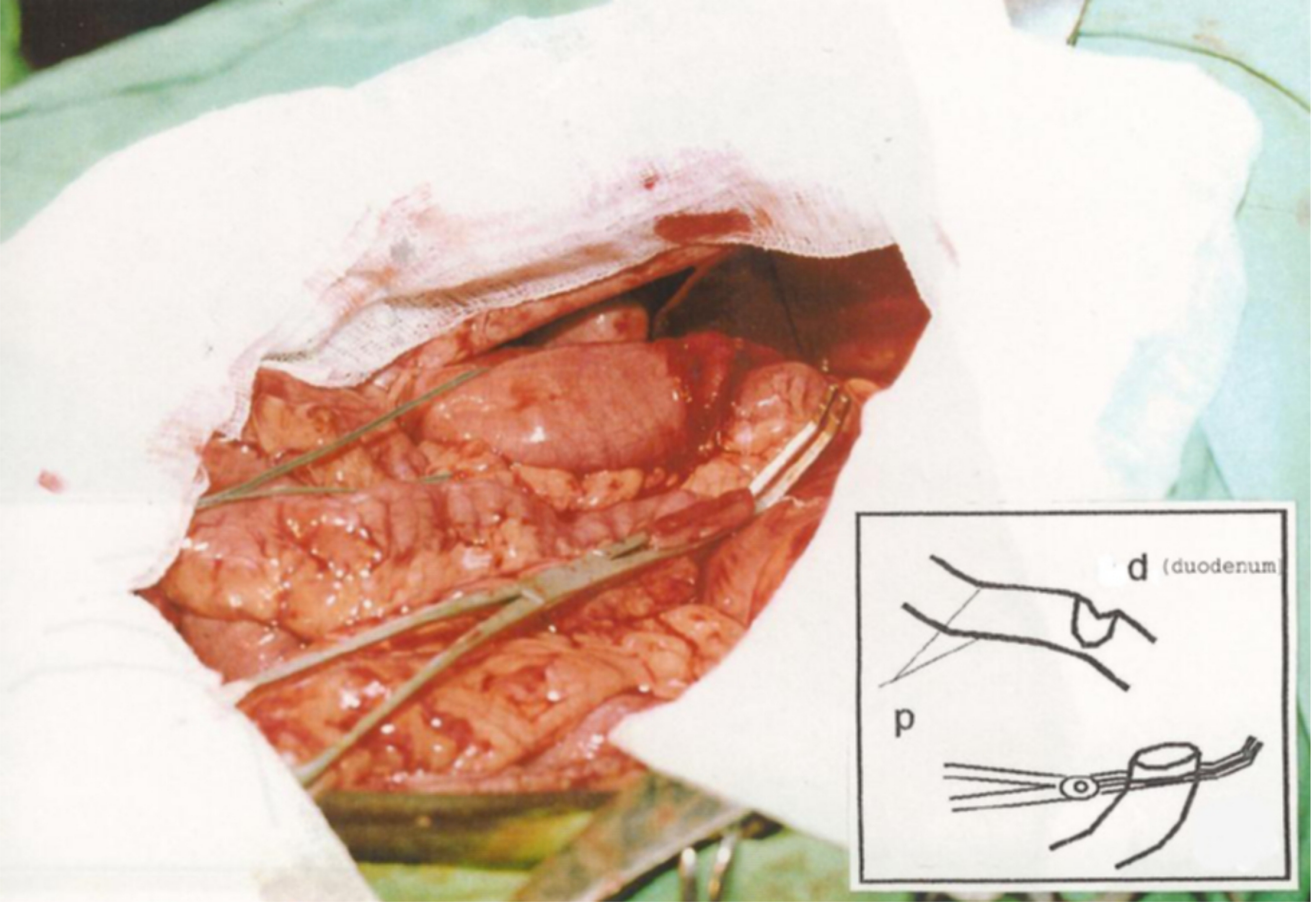
Retrocolic duodeno-jejunal anastomosis side-to-end (the jejunum is closed with a intestinal clamp): “p” = ligature (place of experimentally created intestinal obstruction).

**Fig 4:**
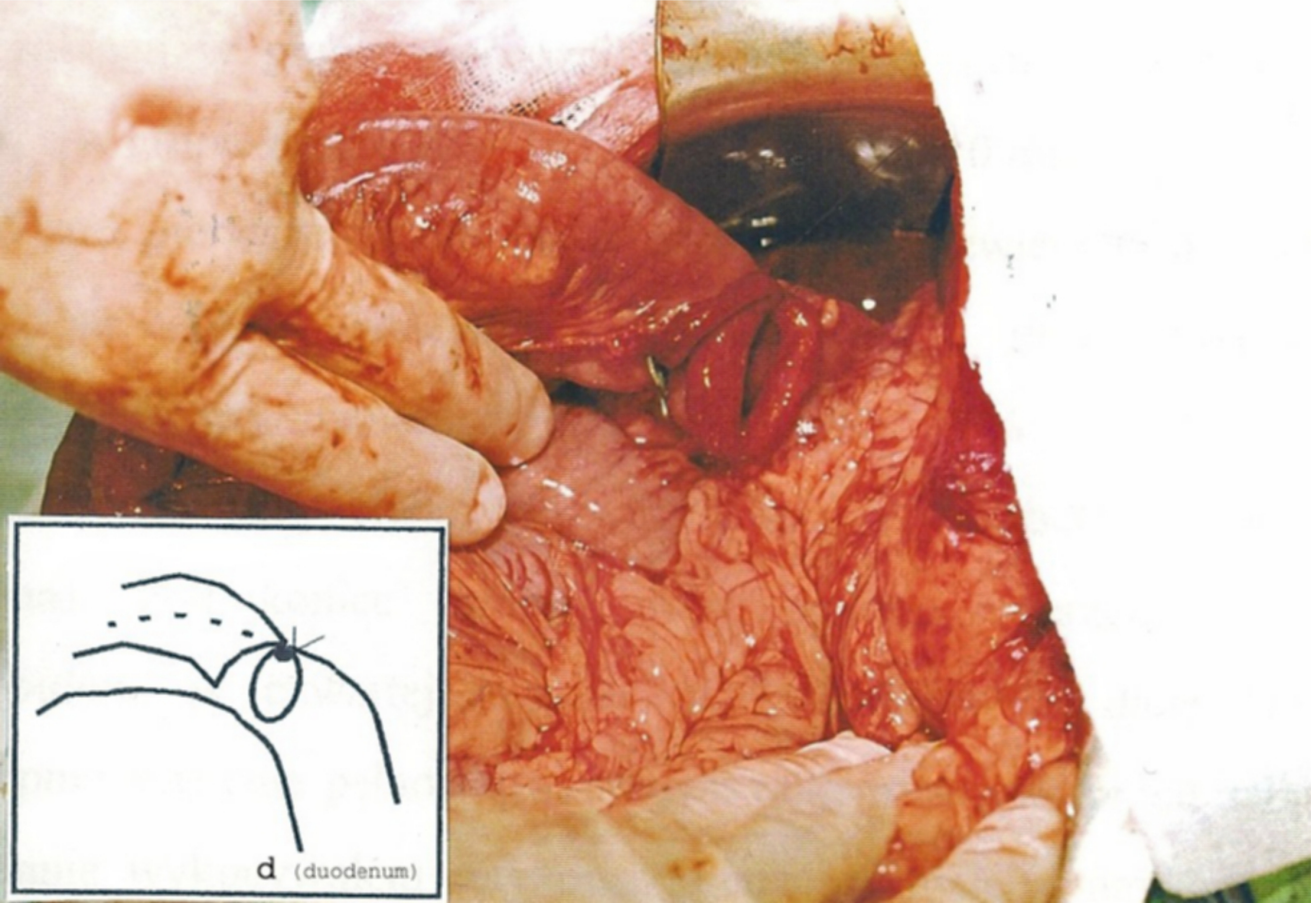
Initial phase of anastomosis creation between the duodenal bulb and the jejunum (4–0 PDS was used for a one layer anastomosis between side of the duodenal bulb and proximal end of jejunum).

In the duodenoanastomosis (DA) group of animals, the creation of an artificial bowel obstruction was achieved using the same technique as described above, although the alimentary canal was opened through an incision of the duodenal bulb wall (Fig 3). Subsequently, Roux-en-Y anastomosis of the side of the duodenal bulb to the end of the first isolated loop of the small intestine was created in one layer using single sutures above the site of intestinal obstruction (Fig 4). The restoration of the continuity of the alimentary canal was conducted through an end-to-side entero-enteric anastomosis in one layer.

The surgery was accomplished within 60–110 min (average 85 min) in the first group, whereas in the second group it lasted 80–140 minutes (average 110 min). The dogs regained access to food on the day following the surgery. Assessment of the welfare of the animals consisted in body mass and complete blood count check accomplished at 7-day intervals. For recovery after the surgery approximately 14 days were allowed.

Thereafter, the animals underwent a series of gastric emptying examinations which were taken during the early (2–3 weeks after surgery), medium (3 months after surgery) and late (6 months after surgery) postoperative periods. At every checkpoint the gastric emptying measurement was performed in duplicate on separate days.

When the research project was finished, the dogs were offered for adoption. Successfully, every animal found its new home and family.

### Radioisotopic examination of gastric emptying

After a 24-hour fast a dog was placed in the modified Pavlov stand and received a test meal consisting of two egg yolks fried until solid on a pan with addition of 5 g butter, and 50 ml milk (7.5 g protein, 5.2 g fat and 2.6 g carbohydrates; total energy content 391 kJ). Before thermal coagulation 1.0 g Amberlite IRA-410 anion-exchange resin (Sigma Chemical Co., USA), labelled with 18.5 MBq (0.5 mCi) ^99m^Tc, was mixed into the raw egg yolks, providing thus a tracer of the solid phase of the meal. The head of a gamma camera (DIACAM, Siemens, Germany) was placed at a distance of 10 cm in parallel to the left side of the dog. The gamma camera detector was equipped with a parallel-hole low energy collimator, and the amplitude analyzer was set to 140 keV with a 15% energy window width. These acquisition parameters enabled a resolution of 4.3 mm per pixel within a 256 x 256 matrix. The registration started immediately after ingestion of the test meal and involved sixty consecutive 3-min counts. The obtained data were stored on a hard disc of a computer for a subsequent off-line analysis.

### Derivation of gastric emptying parameters

A whole stomach region of interest (ROI) was defined by means of an edge finding program. Time activity curves from the gastric ROI were corrected for the physical decay of the isotope and normalized for the fraction of activity remaining within the stomach (considering the maximum activity frame to be 1.0). Correction for the spatial movement of the tracer was ensured by an experimental design considering a dog to be its own control.

The obtained gastric emptying curves were fitted with a power-exponential function in order to obtain two parameters: the gastric emptying half time T½, and S–the curve shape parameter [18,19]:

$$F(t)=100\cdot 2^{-{\left(\frac{t}{T½}\right)}^{S}}$$

where: F(t) = per cent fraction of the radiolabelled meal remaining in the stomach as a function of time, t.

The T½, reflecting the time from the start of the meal until 50% of the meal has emptied, appears to be insufficient to describe quantitatively the time course of gastric emptying. For example, T½ may be identical in extremely different gastric emptying models, such as dumping syndrome with a very rapid initial emptying followed by a second slower emptying phase, as contrasted to a physiological time course of gastric emptying of a solid meal characterized by a curve with an initial lag in emptying and followed by linear gastric emptying phase. In the former case the S would be much less than 1.0, whereas in the latter it would clearly exceed 1.0. The versatility of the power-exponential method was confirmed by the analysis of mathematical curves designed to mimic a range of real life emptying patterns [18,19].

### Statistical methods

Because on every observation point the gastric emptying measurement was taken in duplicate, the results of these two measurements were first averaged and then subjected to statistical analysis. The gastric emptying parameters were compared with the use of the paired or unpaired t test where appropriate. Statistical significance was set at the p<0.05 level, two-tailed. The results are presented as means ± SE. All statistical analyses were performed with the use of Statistica software [20].

## Results

The gastric emptying parameters derived throughout the study are assembled in Table 1, whereas individual results in every dog are provided in S1 Table. It can be inferred therefrom that the basal T½ was slightly longer in the GA compared to the DA group, but the difference was statistically not significant. On the other hand, the basal value of the S parameter was almost identical in the two groups.

**Table 1:** Gastric emptying kinetics of a solid meal (T½ = half emptying time and S = curve shape parameter) before and after two variants of by-pass surgery performed for experimental obstruction of the duodenum: Gastrointestinal anastomosis (GA) or duodenointestinal anastomosis (DA).

	Basal situation	Postoperative period			Differences between the postperative periods and the basal situation		
		Early (2–3 weeks after surgery)	Medium (3 months after surgery)	Late (6 months after surgery)	Early minus Basal	Medium minus Basal	Late minus Basal
GA: **T½** (min)	**228** *±39*	**319** *±26*	**278** *±51*	**244** *±36*	**91** *±37*	**50** *±50*	**17** *±50*
DA: **T½** (min)	**172** *±49*	**154** *±30*	**218** *±51*	**191** *±54*	**-18** *±21*	**46** *±31*	**19** *±61*
Significance of differences GA vs DA	NS	**p = 0.0034**	NS	NS	**p = 0.042**	NS	NS
GA: **S** (dimensionless)	**1.14** *±0*.*12*	**1.66** *±0*.*52*	**1.47** ^**a)**^ *±0*.*05*	**1.79** ^**b)**^ *±0*.*11*	**0.51** *±0*.*51*	**0.32** *±0*.*11*	**0.64** *±0*.*19*
			p = 0.038 vs the basal situation	p = 0.026 vs the basal situation			
DA: **S** (dimensionless)	**1.15** *±0*.*09*	**1.92** *±0*.*52*	**0.85** ^**c)**^ *±0*.*12*	**1.14** *±0*.*15*	**0.77** *±0*.*48*	**-0.30** *±0*.*09*	**-0.01** *±0*.*20*
			p = 0.032 vs the basal situation				
Significance of differences GA vs DA	NS	NS	**p = 0.0031**	**p = 0.0098**	NS	**p = 0.0022**	**p = 0.045**

**Fig 5:**
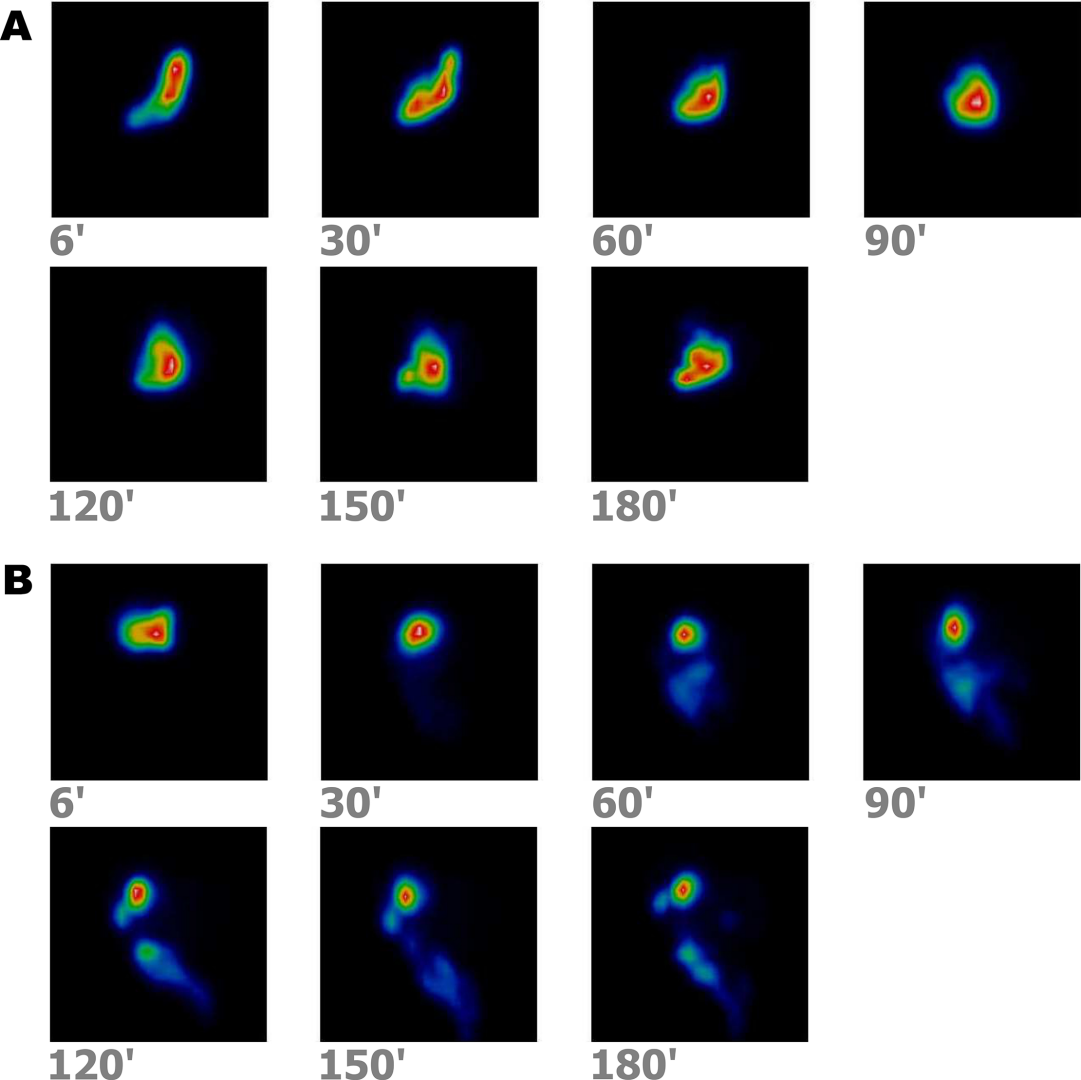
Sequential imaging with a gamma camera of the gastric emptying of a radiolabelled solid meal during the early postoperative period (4 weeks after surgery): series A (upper panel)–in a dog with a gastrojejunoanastomosis (GA), series B (lower panel)–in a dog with Roux-en-Y duodenojejunoanastomosis (DA), created in either case for an experimental obstruction of the duodenum. It can be inferred that radioactivity is retained within the stomach throughout 3 h in the GA dog, whereas a normal passage of the radiolabelled meal from the stomach to the intestines occurs in the DA dog.

**Fig 6:**
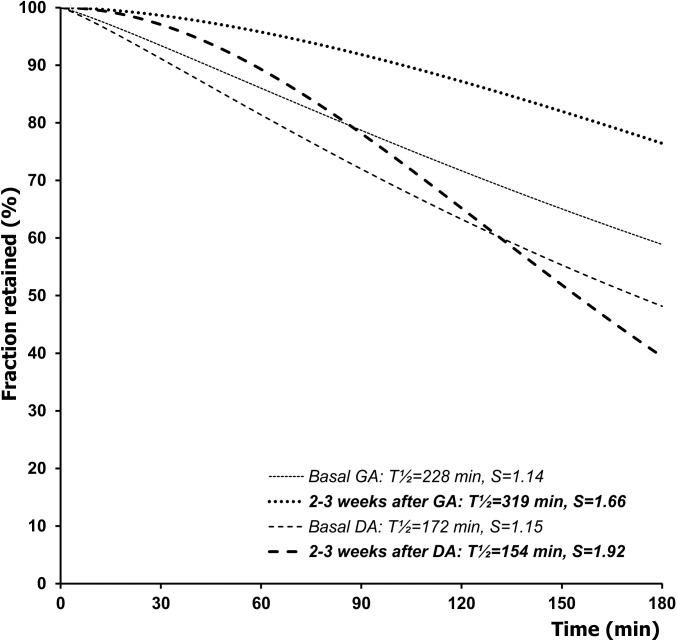
Time course of the gastric emptying of a solid meal, reflected by its fraction retained within the stomach, before surgery and during the early postoperative period (2–3 weeks after surgery) in dogs with a gastrojujenal (GA) or duodenojejunal (DA) anastomosis created for experimental obstruction of the duodenum. The curves were modeled with the use of a power-exponential function computed on group means of T½ and S.

The most striking difference in gastric emptying kinetics between the two groups was found during the early post-operative period (Fig 5) Whereas in the DA group the T½ slightly decreased by a mean of -18±21 min, an opposite shift occurred in the GA group wherein a prolongation of the T½ by a mean of 91±37 min occurred. Thus, the between-group comparison revealed a statistically significant difference either in the case of T½ length (p = 0.0034), or the net change of the T½ relative to the basal value (p = 0.042). In both groups, an increase in the S parameter was found (Table 1). The time courses of gastric emptying curves during the early post-operative period, in agreement with the outlined differences in numerical values of the respective parameters, reflect a considerable delay of gastric evacuation following the GA, whereas in the DA group it remained unimpeded (Fig 6).

**Fig 7:**
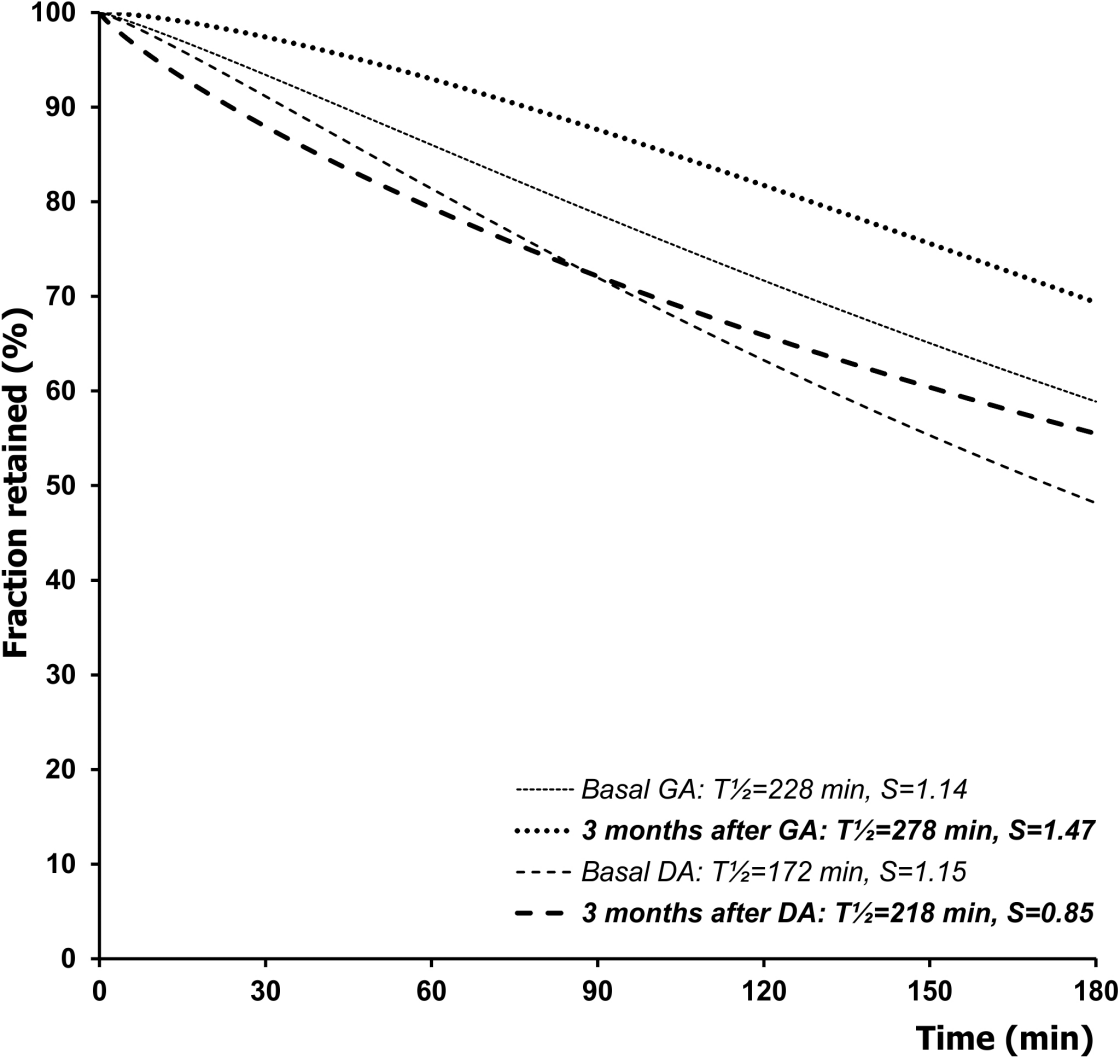
Time course of the gastric emptying of a solid meal, reflected by its fraction retained within the stomach, before surgery and during the medium postoperative period (3 months after surgery) in dogs with a gastrojujenal (GA) or duodenojejunal (DA) anastomosis created for experimental obstruction of the duodenum. The curves were modeled with the use of a power-exponential function computed on group means of T½ and S.

**Fig 8:**
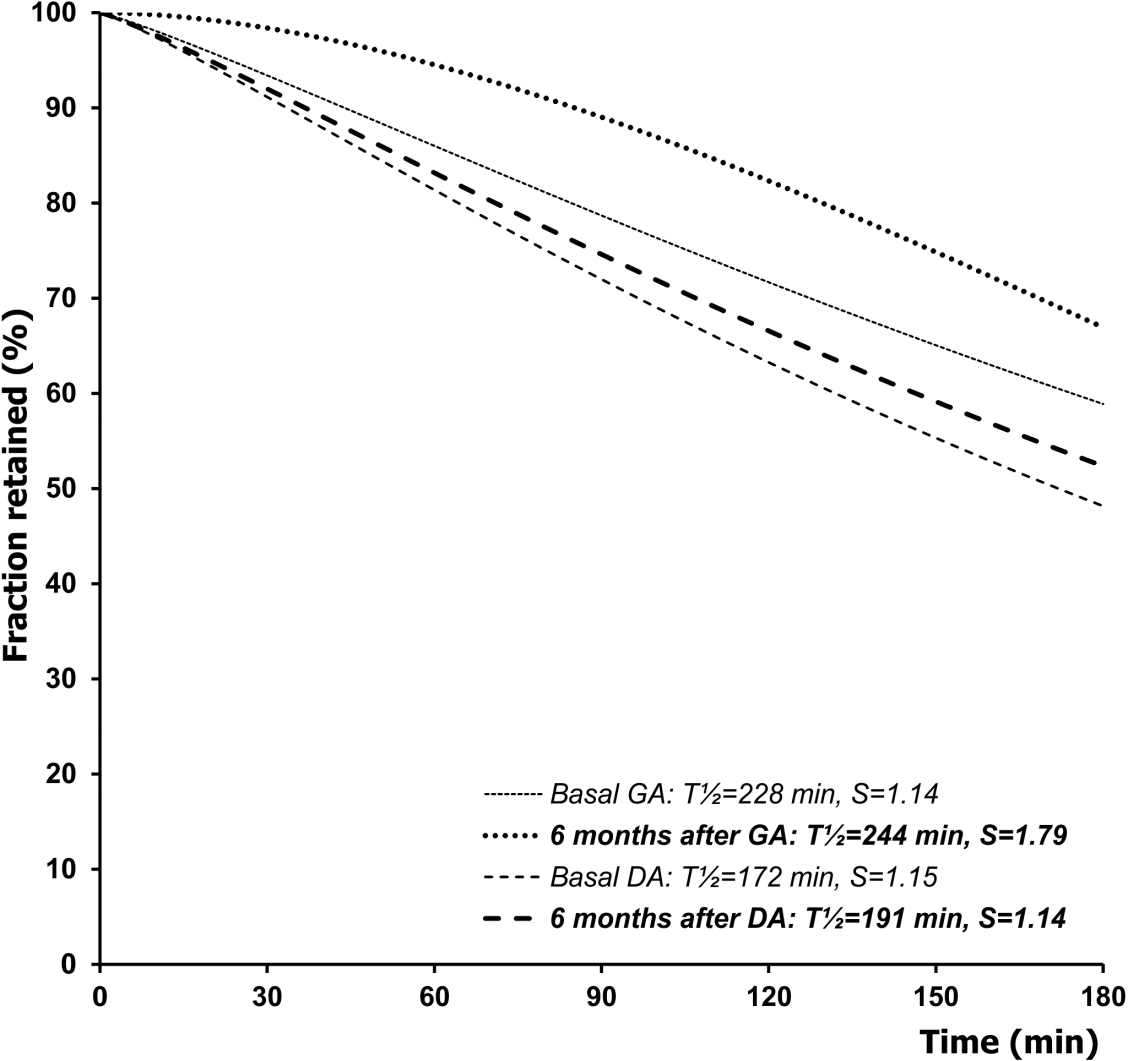
Time course of the gastric emptying of a solid meal, reflected by its fraction retained within the stomach, before surgery and during the late postoperative period (6 months after surgery) in dogs with a gastrojujenal (GA) or duodenojejunal (DA) anastomosis created for experimental obstruction of the duodenum. The curves were modeled with the use of a power-exponential function computed on group means of T½ and S.

In the GA group during the medium and late post-operative period the T½ gradually declined towards the basal value. Similarly, in the DA group at the checkout half a year after the surgery the T½ nearly approached the pre-operative value. A considerable difference was, however, revealed with regard to the S parameter. In the GA group it remained to be augmented throughout the medium and the late postoperative period, whereas in the DA group–quite opposite–it continued to decline and finally after a lapse of 6 months it equaled the basal value. Consequently, the net differences relative to the basal situation in the S values appeared to be positive in the GA group (0.32±0.11 at 3 months and 0.64±0.19 at six months), and negative in the DA group (-0.30±0.09 at 3 months and -0.01±0.20 at six months). Hence a statistically significant between-group contrast was found between those differences: p = 0.0022 at 3 months, and p = 0.045 at six months after the surgery (Table 1). Comparison of the relevant curves indicates that after the DA surgery the time course of gastric emptying was very close to the basal situation, whereas in the other group considerable retention of the radiolabelled meal within the stomach occurred either three months (Fig 7) or six months (Fig 8) after creation of the GA.

## Discussion

The diagnosis of pancreatic cancer is usually delayed–in patients who undergo a surgical reconnaissance a highly advanced regional pancreatic cancer is frequently revealed, which is a finding definitely limiting resectability (10–20% of the patients). Despite the progress that has been made recently in the field of laparoscopic surgery and endoscopic stent therapy for palliative management of pancreatic cancer, surgical intervention remains the treatment of choice when the diagnostic laparotomy reveals that radical resection is not feasible or contraindicated [21,22]. Surgery is indicated particularly for patients with predicted long-term survival [22]. Recent reports indicate also that routine palliative bypass is recommended for palliation in patients with adenocarcinoma of the pancreas who had been explored with a curative intent but turned out to have an inoperable disease stage discovered at the time of surgery [23,24].

Other authors found that a prophylactic gastrojejunostomy significantly decreased the incidence of gastric outlet obstruction in patients with unresectable periampullary cancer [25,26]. Accordingly, a quite common complication after a gastric bypass is a delay of GE, the incidence of which ranges between 2 and 14% [26]. According to Kajiwara et al. [27] an optimal method of reconstruction in such cases is the duodeno-jejunal end-to-end method. The implementation of the anastomosis at the upper part of the duodenum was not described formerly. Nevertheless, Bloodgood in 1907 was the first to suggest duodenojejunostomy as a method of surgical management, which was later (1921) adopted as a procedure for Wilkie’s syndrome (the superior mesenteric artery syndrome). He concluded that this procedure was the treatment of choice in this syndrome resulting from the compression of the third portion of the duodenum between vessels, spine and the paravertebral muscles [28,29]. Hence the use of the first part of the duodenum as the site of the anastomosis appears to be an alternative method of anastomosis with the stomach.

The regulation of GE is a concerted function of the stomach, pylorus and the duodenum which act as a functional motoric unit [30]. According to Wyse et al. [31], GE of solids does have a greater clinical significance because disordered GE of liquids is detected rather occasionally. A disordered GE may contribute to postoperative complications which may occur just early but not infrequently also during the late postoperative period. Therefore, a clinically sound survey should comprise repeat examinations of GE over a prolonged period of time. Helpful in this respect is experimental surgery in animal models which enables monitoring of GE kinetics over time, permitting thus to reveal and compare either early or late postoperative sequleae of variant surgical procedures [32–37]. No doubt the canine model has been for a long time preferred in the examinations of gastric emptying. Postoperative gastric emptying disorders in dogs involving the disruption of the stomach anterior wall was reported by Mistiaen et al. [38] who using a radioisotopic technique showed a significant slowdown of gastric evacuation two weeks after the operative procedure. A co-ordination between the canine gastric and duodenal activity was reported by Ehrlein and Hiesinger [39]. Keinke et al. [40] reported next on regulatory factors involved in gastric emptying in dogs which include: antral peristaltic contractions, opening of the pylorus and relaxant sensitivity of the duodenum. According to Miller et al. [41] normal gastric relaxation seems to be one of the most important factors influencing gastric emptying. An in-depth report on the relationship between pressure adaptation of the fundus and the body of the stomach and pressure variations during meal passage with a possibility to evoke a delayed gastric evacuation was reported by Keinke et al. [40].

Admittedly, nowadays there exists a trend to examine gastric emptying with methods not involving radiation, such as ultrasonography or ^13^C-octanoic acid breath test (^13^C-OABT)–an attitude, which is entirely justified with regards to human studies. Such a limitation does not pertain animal studies. There is a common agreement that scintigraphy remains a “golden standard” with regard to the measurement of gastric emptying speed. It enables monitoring of stomach emptying in real-time, as opposed to indirect methods, such as ^13^C-OABT [42,43].

Taking into account the above, we decided to use the scintigraphic measurement of the gastric emptying of a ^99m^Tc-labelled solid meal in our study–bearing in mind during its conduct technical hints provided by other authors–methodology similar to ours was formerly used by Chiba et al. [44], who also applied ^99m^Tc-labelled Amberlite 410 resin pellets as a marker of the gastric emptying of a solid meal. Also, a process of training the dogs to get them adapted to a Pavlov stand was pursued by the cited authors.

Our study was devoted to compare the functionality of a routinely performed surgical anastomosis between the stomach and the intestine with an anastomosis created between the proximal part of the duodenum and the intestine, applied in both cases for an artificial occlusion of the distal duodenum. The results revealed a significantly better gastric evacuatory function during the post-operative survey after Roux–en-Y when compared to a classical method of a gastrojejunal anastomosis. The striking advantage of Roux-en-Y procedure over the traditional approach was observed especially during the early post-operative period. It seems that a clue for the explanation of this finding provide studies which sought for consequences of gastric transection. It was shown that a slit between the body and the pre-pyloric region of the stomach causes disruption of slow wave propagation pathways from the stomach body to the antrum [45]. As is well known, the gastric slow waves constitute an electrical basis for a proper contractile activity of the stomach. Hence, a postoperative sequel in the case of an anastomosis connected to the stomach body or antrum would consist in decreased pyloric contractions and delayed emptying of solid meals [46–48]. On the other hand, creation of an anastomosis below the pylorus, for example its connection to the first part of the duodenum, which was the case in our study, should leave the interplay of the stomach electrical and motor activity unaffected. Indeed, a hint with respect to correctness of this reasoning was formerly given by Chiyasate et al. [34]. In a study conducted in dogs they proved that reconstruction with an uncut Roux limb and jejunal pouch after total gastrectomy preserved a unidirectional migration of intestinal myoelectrical activity, what ultimately resulted in an improvement of postoperative weight gain and nutritional parameters of the animals during a 10-week observational period [34].

One should be aware that in real-life situation a choice between variant modes of by-passing operations may be strongly affected by the local growth of a pancreatic tumour. In the case of high gastrointestinal obstruction in humans, we suggest considering the DA anastomosis primarily for an unresectable tumour of the peripheral part of the pancreas, coming out of the neck or the body or the pancreas and blocking the lower and the horizontal part of the duodenum. In the case of an unresectable tumour of the head of the pancreas, the indications for this type of bypass should be considered individually. Some of these bulky unresectable pancreatic head masses originate from the lower part of the head of the pancreas and the uncinate process. In such a case, the duodenal bulb remains free and one can then consider an anastomosis between it and the intestine. The anastomosis could be done side-to-side or by connecting the end of the intestine to the side of the duodenal bulb. On the other hand, the DA anastomosis cannot be performed in patients who have a displaced jejunum loop or when the connection to the duodenum is close to the tumour. This method of anastomosis is an alternative solution to the classic GA in cases where the duodenal bulb and the path of the loop moved to create the anastomosis are significantly distant from the tumour.

Summing up, frequently observed disturbances of gastric emptying after gastroenterostomy impel to seek new ways to treat duodenal obstruction. One of such methods is the anastomosis of the duodenal bulb with the intestine. This type of anastomosis is not always possible when the stenosis is close to the pylorus. However, if the stenosis is located below the horizontal part of the upper duodenum, the duodenal bulb can be used as a potential connection site. The purpose of this procedure is to preserve the anatomical integrity of the stomach walls, including innervation. The results obtained in the presented study indicate potential benefits for patients. Therefore, it seems advisable to undertake a randomized clinical trial comparing GA and DA in a representative cohort of patients.

## Conclusion

The results of our study indicate that thanks to its favourable effect on the efficiency of solid phase gastric emptying, especially during the early post-operative period, the Roux-en-Y duodenojejunal anastomosis appears to be superior to the classical gastrojejunal anastomosis while restoring patency of gastrointestinal passage in case of an upper bowel obstruction.

## Supporting information

S1 Table IGED.xlsx. Table: IndividualGastricEmptyingData.
(XLSX)
